# Expression of *LGR5* in mammary myoepithelial cells and in triple-negative breast cancers

**DOI:** 10.1038/s41598-021-97351-y

**Published:** 2021-09-07

**Authors:** Hyun Ju Lee, Jae Kyung Myung, Hye Sung Kim, Dong Hui Lee, Hyun Su Go, Jae Hyuck Choi, Hyun Min Koh, Su-Jae Lee, Bogun Jang

**Affiliations:** 1 Department of Pathology, Soonchunhyang University College of Medicine and Soonchunhyang University Cheonan Hospital, Cheonan, Korea; 2 Department of Pathology, Hanyang University College of Medicine, Seoul, Korea; 3 Department of Pathology, Jeju National University School of Medicine, Jeju, South Korea; 4 Department of Surgery, Jeju National University School of Medicine and Jeju National University Hospital, Jeju, South Korea; 5 Department of Pathology, Gyeongsang National University Changwon Hospital, Changwon, South Korea; 6 Department of Life Science, Research Institute for Natural Sciences, Hanyang University, Seoul, South Korea; 7 Department of Pathology, Jeju National University School of Medicine and Jeju National University Hospital, Aran 13 gil 15, Jeju city, Jeju, 63241 Korea

**Keywords:** Cancer, Breast cancer

## Abstract

Lineage tracing in mice indicates that *LGR5* is an adult stem cell marker in multiple organs, such as the intestine, stomach, hair follicles, ovary, and mammary glands. Despite many studies exploring the presence of *LGR5* cells in human tissues, little is known about its expression profile in either human mammary tissue or pathological lesions. In this study we aim to investigate *LGR5* expression in normal, benign, and malignant lesions of the human breast using RNA in situ hybridization. *LGR5* expression has not been observed in normal lactiferous ducts and terminal duct lobular units, whereas *LGR5*-positive cells have been specifically observed in the basal myoepithelium of ducts in the regenerative tissues, ductal carcinoma in situ, and in ducts surrounded by invasive cancer cells. These findings suggest *LGR5* marks facultative stem cells that are involved in post injury regeneration instead of homeostatic stem cells. *LGR5* positivity was found in 3% (9 of 278 cases) of invasive breast cancers (BC), and it showed positive associations with higher histologic grades (*P* = 0.001) and T stages (*P* < 0.001), while having negative correlations with estrogen receptor (*P* < 0.001) and progesterone receptor (*P* < 0.001) expression. Remarkably, all *LGR5*-positive BC, except one, belong to triple-negative BC (TNBC), representing 24% (9 of 38 cases) of all of them. *LGR5* histoscores have no correlations with EGFR, CK5/6, Ki-67, or P53 expression. Additionally, no β-catenin nuclear localization was observed in *LGR5*-positive BC, indicating that canonical Wnt pathway activation is less likely involved in *LGR5* expression in BC. Our results demonstrate that *LGR5* expression is induced in regenerative conditions in the myoepithelium of human mammary ducts and that its expression is only observed in TNBC subtype among all invasive BC. Further studies regarding the functional and prognostic impact of *LGR5* in TNBC are warranted.

## Introduction

Leucine-rich repeat containing G-protein-coupled receptor 5 (*LGR5*) encodes a seven-transmembrane receptor belonging to the G-protein-coupled receptor rhodopsin family. *LGR5* and its close homologs, *LGR4* and *LGR6*, are potent enhancers of canonical Wnt/β-catenin signaling by binding to secreted R-spondin growth factors^1^. In the absence of R-spondins, the E3-ubiquitin ligases Rnf43/Znrf3 degrades the Frizzled receptor, leading to downregulation of Wnt signaling^2^. As Rnf43/Znrf3 are themselves transcriptional Wnt/β-catenin signaling targets, they serve as components of a negative Wnt feedback loop^2^. *LGR5* has been identified as a homeostatic stem cell exquisite marker in various tissues, including the intestines, stomach, hair follicles, ovaries, and mammary glands^3–7^. Subsequently, *LGR5* + cells have also been demonstrated to be facultative stem cells responsible for postinjury regeneration in the liver, pancreas, and stomach^8–10^. Homeostatic *LGR5* + stem cells contribute to various cancers such as colorectal cancers, gastric cancers, and squamous cell skin carcinomas when oncogenic mutations occur^11–13^.

Cancer stem cells are widely believed to be responsible for cancer initiation and progression. They are a small tumor population with stem cell properties. A growing number of studies demonstrate that CSCs are remarkably heterogeneous and plastic. Therefore, they can convert from differentiated cells under permissive conditions^14^. In colorectal cancers, *LGR5* + cells have been demonstrated to act as cancer stem cells fueling tumor growth and metastasis^15,16^. In addition, Yang et al. suggested that *LGR5* plays a key role in maintaining breast cancer (BC) stem‐like cells through Wnt/β‐catenin signaling^17^. There also exist several studies that have examined the prognostic significance of *LGR5* in BC, and mostly they show an immunohistochemical staining to detect *LGR5*^+^ cells in cancer tissues^17–19^. However, it is well known that there are no reliable antibodies for marking *LGR5* + cells with formalin-fixed paraffin-embedded (FFPE) tissues. Recently RNA in situ hybridization (ISH) techniques have been used to visualize *LGR5* + cells in human tissues, and this has been proven to be successful in many types of cancers. For BC, Ogasawara et al. have demonstrated specific *LGR5* mRNA expressions using an RNAscope in 43 tripe negative BC^20^. In this study, we aim to thoroughly investigate *LGR5* expression in a large number of pathologic breast lesions, including not only invasive cancers but also a variety of benign lesions.

## Material and methods

### Subjects

We obtained BC tissues from 293 patients (278 invasive carcinoma and 15 DCIS cases) who had undergone surgical resection at Jeju National University Hospital between 2012 and 2019. We gathered clinical pathological information, including age, gender, size, tumor grade, presence of lymphovascular invasion, lymph node metastasis, American Joint Committee on Cancer/International Union against Cancer (AJCC/UICC) cancer staging (7th edition), and positivity for ER, PR, CK5/6, EGFR and HER2 from the patients’ medical records. BCs were subclassified according to ER, PR, and HER2 expression, luminal A, luminal B, HER2, and TNBCs. We also collected normal tissues and benign mammary lesions, including normal lobules (*n* = 5), lactiferous ducts (*n* = 5), fibroadenomas (*n* = 7), phyllodes tumors (*n* = 2), intraductal papillomas (*n* = 3), adenoses (*n* = 5), and inflammatory (*n* = 7) or post-biopsy or excision tissues (*n* = 3). This study was approved by the ethics committee of the Institutional Review Board of Jeju National University Hospital (IRB No.: 2019-04-006, “Expression analysis of LGR5 in breast cancer”) and was conducted in accordance with the Declaration of Helsinki. Informed consent from the patients was waived with IRB approval.

### Tissue microarray construction

In total, 16 tissue microarrays (TMAs) were constructed from archival FFPE tissue blocks, including 293 primary BC tissues and 37 benign lesions. In brief, through histologic examination, a representative tumor portion was carefully selected from hematoxylin- and eosin-stained slides. Each tumor area comprised more than 70% of the cell population. The 4-mm diameter core tissues were obtained from individual BC paraffin blocks or benign lesions and arranged in a new recipient paraffin block (tissue array block) using a trephine apparatus (SuperBioChips Laboratories, Seoul, Korea).

### Immunohistochemistry and interpretation

Immunohistochemistry (IHC) was done with the Ventana Benchmark Ultra platform (Ventana Medical Systems Inc., Tucson, AZ, USA); estrogen receptor (ER) (clone, SP1; Cat. No., 790-4324), progesterone receptor (PR) (clone, 1E2; Cat. No., 790-2223), HER2 (clone, SP3; Cat. No., 790-4493), CK5/6 (clone, D5&16B4; Cat. No., 790-4554), EGFR (clone, 3C6; Cat. No., 790-2988), P53 (clone, D0-7; Cat. No., 800-2912), P63 (clone, 4A4; Cat. No., 790-4509) and Ki-67 (clone, 30-9, Cat. No., 790-4286). HER2 expression was scored according to the 2007 ASCO/CAP guidelines: 0, no staining; 1 +, weak and incomplete membranous staining in ≥ 10% of the tumor cells; 2 +, weak-to-moderate complete membranous staining in ≥ 10% of the tumor cells; and 3 + , strong, complete membranous staining in ≥ 30% of the tumor cells^21^. HER2 was defined as positive when the IHC score is 3 or fluorescent in situ hybridization (FISH) is positive for the cases with IHC score 2. ER and PR were scored with the Allred system (range: 0–8); defined as being positive when it is more than 3. The intensity and percentage of EGFR and CK5/6 tumor cell expressions were measured by multiplying the intensity score (0 = negative; 1 = weak; 2 = moderate; 3 = strong) and percentage of positive cells (range = 0–100), ranging from 0 to 300. P53 and Ki-67 staining was recorded as the percentage of nuclear stained tumor cells. IHC for β-catenin was performed using a BOND-MAX automated immunostainer and a Bond Polymer Refine Detection kit (Leica Microsystems, Wetzlar, Germany) (clone, 17C2; Novocastra Laboratories, Newcastle, UK), and nuclear was considered as positive when more than 10% of tumor nuclei were stained.

### *LGR5* RNA in situ hybridization

We performed *LGR5* mRNA detection using an RNAscope kit (Advanced Cell Diagnostics, Hayward, CA, USA) with unstained tissue slides according to the manufacturer's instructions. Tissue sections were pretreated with protease application and heating prior to hybridization with an *LGR5*-specific probe. The detailed procedure is described in an earlier publication^17^. Brown punctate dots present in the nucleus and/or cytoplasm indicated positive staining. *LGR5* expression was quantified according to the five-grade scoring system recommended by the manufacturer (grade 0: no staining, 1: grade 1–3 dots/cell, grade 2: 4–10 dots/cell, grade 3: > 10 dots/cell, grade 4: > 15 dots/cell with > 10% of dots in clusters). The grade and percentage of tumor cells expressing *LGR5* were measured, and histoscores (H-scores) were calculated by multiplying the grade (range = 1–4) and percentage of *LGR5*-positive tumor cells (range = 0–100), ranging from 0 to 400. For statistical analyses, the case was defined as being positive if H-scores are more than 10. For dual ISH for *LGR5* and IHC for P63, IHC was conducted after completion of the in situ hybridization protocol.

### Statistics

The SPSS (Statistical Package for the Social Sciences) statistical software version 18.0 (SPSS, Chicago, IL, USA) and Prism version 9.0.1 (GraphPad Software, San Diego, CA, USA) were used for analysis. We compared *LGR5* H-scores between subtypes of invasive BC by using Tukey’s Multiple Comparison Test. We analyzed the *LGR5* positivity clinical correlation study with the Pearson χ^2^ test. The correlations between the *LGR5* H-scores and several molecular marker expressions were evaluated by the Spearman correlation test. Differences were considered significant when *P* < 0.05.

## Results

### *LGR5* expression in normal breast lobules, benign lesions, and ductal carcinoma in situ

**Figure 1:**
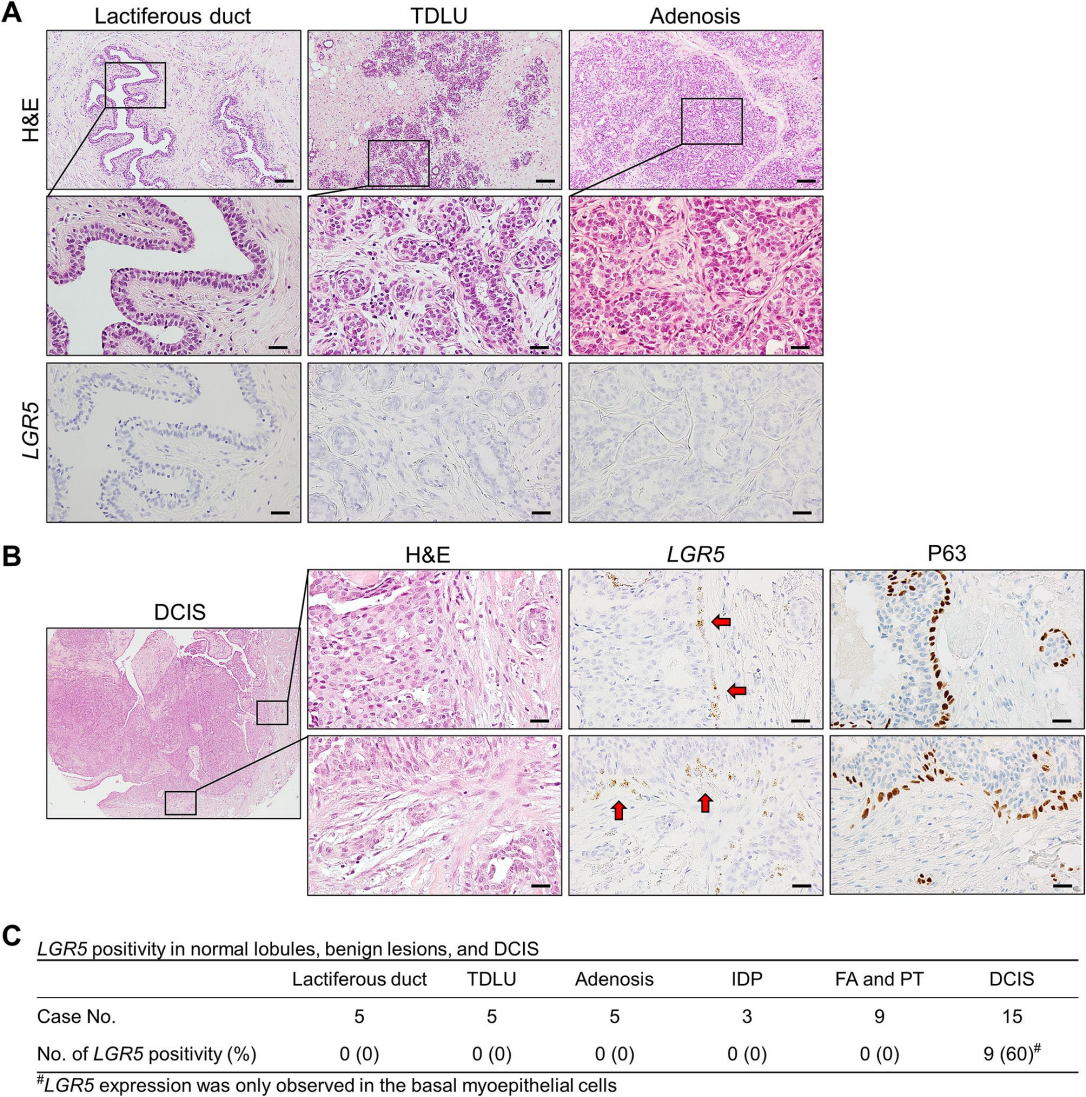
*LGR5* Expression in Benign Lesions and Ductal Carcinoma In Situ (DCIS). (**A**) No *LGR*5 expression was observed in lactiferous ducts, terminal duct lobular unit, and adenosis. (**B**) Representative images of *LGR5* expression in DCIS. Red arrows indicate *LGR5*-positive myoepithelial cells that express P63. (**C**) A table showing the percentages of *LGR5* positivity in benign lesions and DCIS. *H&E* hematoxylin and eosin; *No* number; *TDLU* terminal duct lobular unit; *IDP* intraductal papilloma, *FA* fibroadenoma; *PT* phyllodes tumor. Scale bars: 50 μm.

*LGR5*^+^ cells have consistently been observed in the basal myoepithelial cells of murine mammary ducts near the nipple^7,22–24^. To see whether *LGR5-*expressing cells exist in proximal human breast ducts, we collected five cases of lactiferous ducts. However, *LGR5* expression was not observed in either the luminal or the basal cells (Fig. 1A). We investigated *LGR5* expression in normal terminal duct lobular units (TDLU) and various benign lesions, including adenosis, intraductal papillomas, fibroadenomas, and phyllodes tumors. None of them showed *LGR5* expression. Next, we examined preinvasive BC (15 DCIS cases) and *LGR5* expression was focally observed in 9 cases (60%). Notably, *LGR5* expression was completely restricted to the basal cells surrounding the DCIS, whereas no carcinoma cells expressed *LGR5* (Fig. 1B and Supplementary Fig. S1). The overall positivity rates are summarized in Fig. 1C. These findings suggest that normally there are no *LGR5*-positive cells in adult human mammary ducts and lobules, but basal cells expressing *LGR5* emerge in DCIS.

### *LGR5* induction in myoepithelial cells of the regenerative mammary ducts

**Figure 2:**
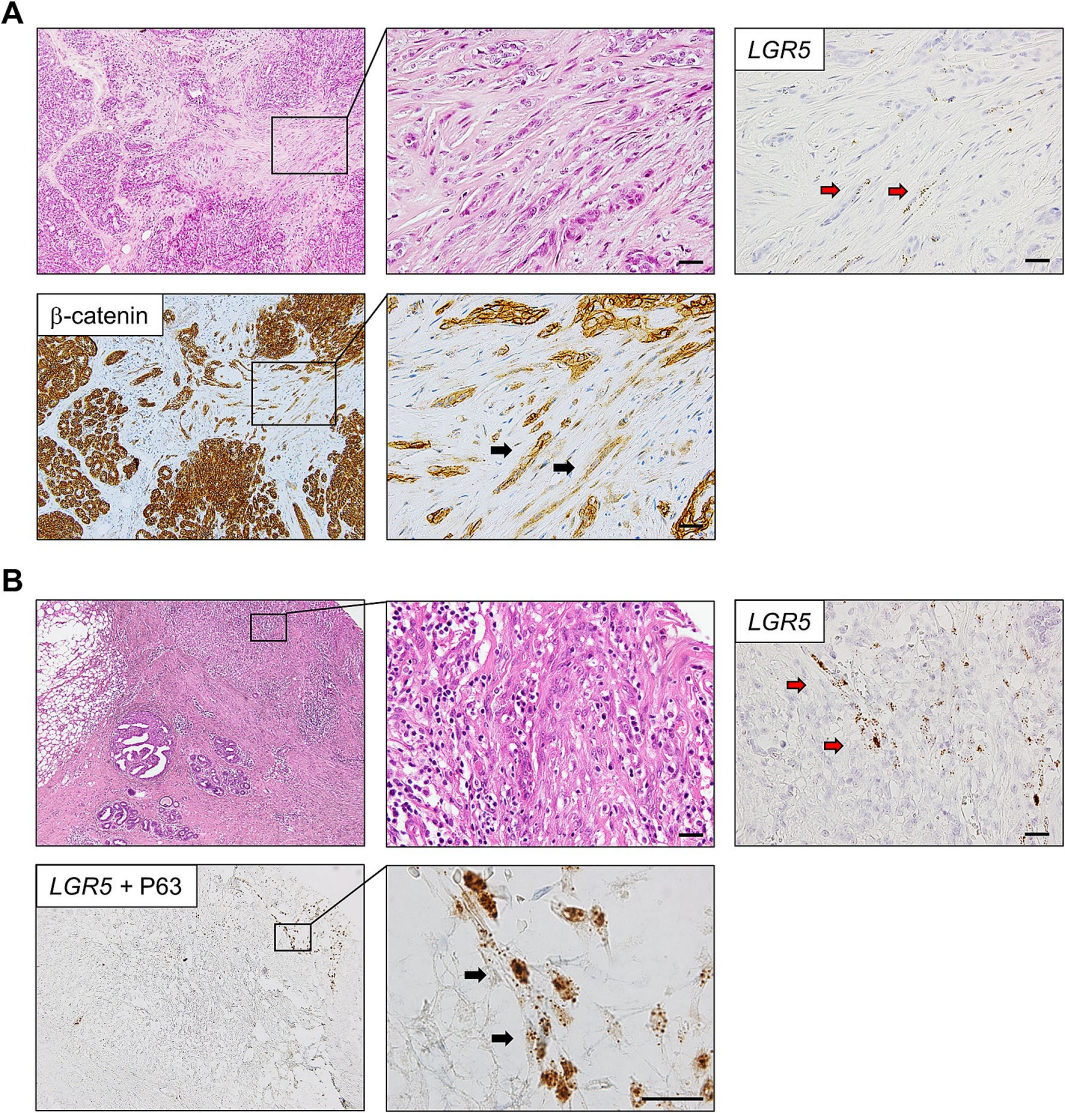
Induced *LGR5* Expression in Regenerative Tissues. (**A**) RNA in situ hybridization showed the *LGR5*-positive cells in the needle biopsy-induced scar area in adenosis (indicated by red arrows) and β-catenin staining confirmed that they are epithelial cells (indicated by black arrows). (**B**) Regenerative areas after excisional biopsy shows a group of *LGR5*-positive cells mixed with inflammatory cells. Dual staining for P63 (brown nuclear stain) and *LGR5* (brown dots in the cytoplasm) demonstrated *LGR5*-expressing cells are myoepithelial cells that are positive for P63 (indicated by black arrows). Scale bar: 50 μm.

*LGR5* cells have been identified as reserve stem cells in several adult murine organs, but only upon tissue injury for recovery. As *LGR5* cells are not present in normal and benign mammary lesions, we explored whether they could be induced under regenerative conditions. Among the 10 inflammatory or healing lesions examined, we found two cases where *LGR5* cells emerged in regenerative ducts. The first case was an excisional specimen of adenosis containing a scar area induced by the needle biopsy. We observed *LGR5*-positive cells in the linear ductal structures and β-catenin staining demonstrated that they were epithelial cells but not stromal cells (Fig. 2A). The other was a resected specimen where *LGR5*-positive cells were observed in the inflamed ducts around the excision site (Fig. 2B). To identify what type of cells express *LGR5*, we performed dual stain for *LGR5* and P63 on the second case, and we confirmed that *LGR5* cells are P63-positive myoepithelial cells (Fig. 2B). These findings suggest that in human mammary tissues, *LGR5* cells can emerge under certain conditions such as regeneration following tissue injury.

### *LGR5* expression in four subtypes of invasive breast cancers

**Figure 3:**
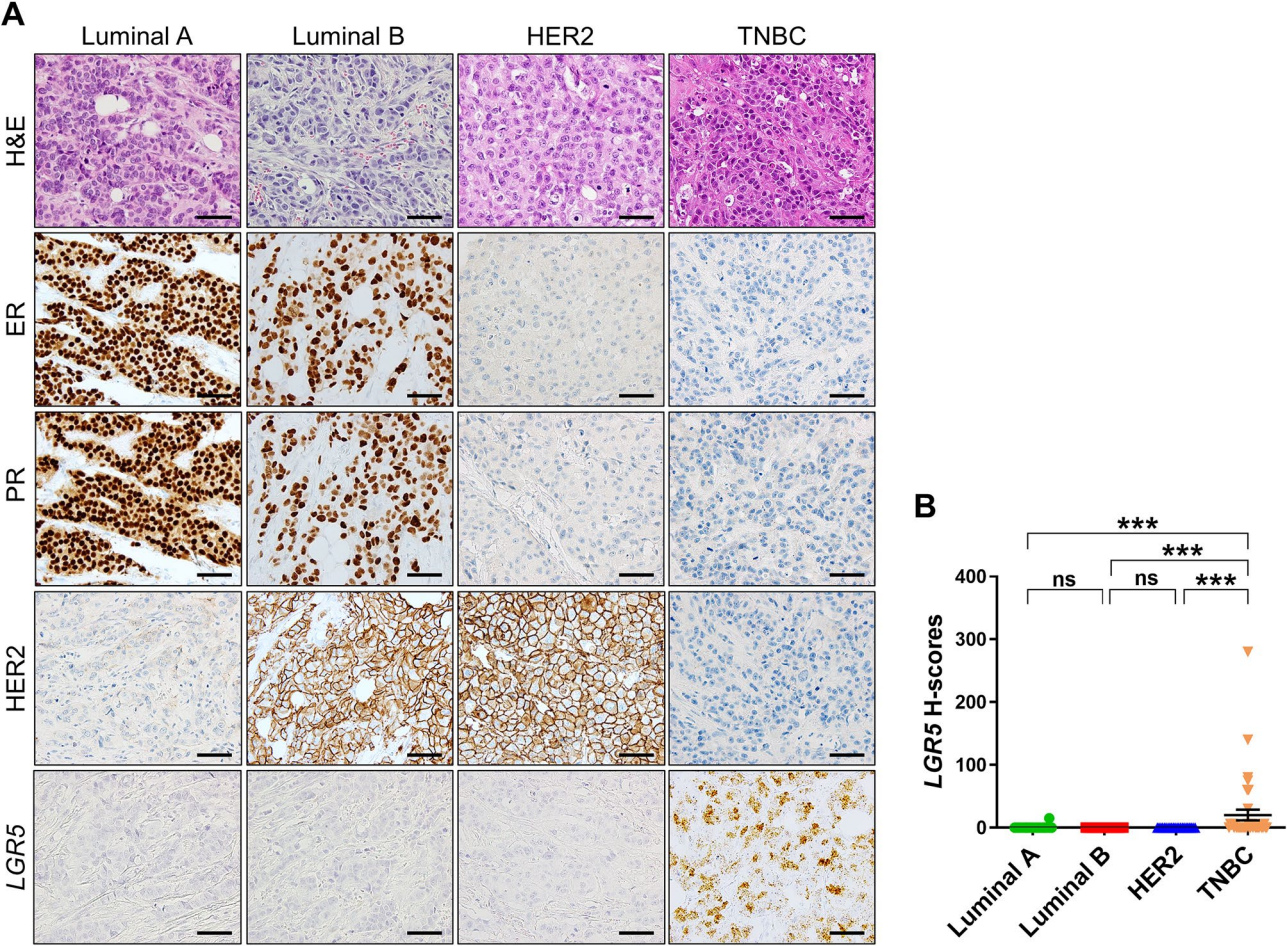
*LGR5* Expression in Invasive Breast Cancers of Four Molecular Subtypes. (**A**) Representative H&E staining, immunohistochemical staining (ER, PR, and HER2), and in situ hybridization (*LGR5*) according to four subtypes of breast cancers. (**B**) A graph showing histoscores of *LGR5* in BCs. ER, estrogen receptor; PR, progesterone receptor; TNBC, triple negative breast cancer. Ns, not significant. ***P < 0.001. Scale bars: 100 μm.

We measured *LGR5* H-scores in a large cohort of invasive BC (*n* = 279) and a total of 18 cases of *LGR5*-expressing BC were observed. The pathological features of them are shown in Table 1, and 9 cases with an H-score of 10 or higher were considered positive for statistical analysis. Interestingly, in some cases, we observed a remarkable increase in *LGR5* expression in the myoepithelium of nonneoplastic ducts surrounded by cancer cells (Supplementary Fig. S2). The associations between *LGR5* positivity and clinicopathological characteristics are summarized in Table 2. Histologically, *LGR5* expression was only observed in invasive carcinomas of no special type. It was associated with poor tubule formation, marked nuclear pleomorphism (*P* < 0.001), and a high mitotic count (*P* < 0.001). Thus, it was not surprising to find that all *LGR5*-positive BC were scored as grade 3 (*P* < 0.001). *LGR5* expression was more frequently observed in BC with higher T stages (*P* < 0.001), whereas there were no correlations with lymphovascular invasion (*P* = 0.428), N stages (*P* = 0.748), or AJCC (7th edition) tumor stages (*P* = 0.545). Based on ER, PR, and HER2 positivity, BCs were classified into four molecular subtypes: luminal A, luminal B, HER2, and TNBC. Interestingly, *LGR5* positivity showed strong negative correlations with ER (*P* < 0.001) and PR (*P* < 0.001) expressions, and it turned out that all *LGR5*-positive BCs except one belonged to the TNBC subtype (*P* < 0.001), comprising 21% of all TNBCs (8 out of 38 cases). Representative images, including H&E stain, immunohistochemical stain for ER, PR, and HER2, and in situ hybridization for *LGR5* are shown in Fig. 3A. When comparing *LGR5* H-scores between molecular subtypes, they were significantly higher in TNBC than in other types (Fig. 3B). As *LGR5* is one of the Wnt target genes, we additionally explored whether Wnt/b-catenin signaling activity is responsible for *LGR5* expression in TNBC by evaluating the nuclear expression of b-catenin, indicative of upregulated Wnt signaling. However, none of the *LGR5*-positive BCs showed nuclear b-catenin positivity (Supplementary Fig. S3).

**Table 1:** Clinicopathological characteristics of *LGR5*-expressing invasive breast cancers.

Case	Age	Size (cm)	Histology	TD	NP	MC	Grade	LVI	T	N	Stage	ER	PR	HER2	Molecular Subtype	*LGR5* H-scores	Ki-67 (%)	P53 (%)	EGFR H-scores	CK5/6 H-scores
1	65	5	Inv Ca NST	3	3	3	3	P	2	1a	2b	0	0	0	TNBC	3	80	70	60	0
2*	65	1.5	Inv Ca NST	3	3	3	3	N	1c	0	1a	0	0	0	TNBC	30	96	98	160	270
3	45	5	Inv Ca NST	3	3	2	3	N	3	0	2b	0	0	0	TNBC	6	30	40	30	6
4*	51	7	Inv Ca NST	3	3	3	3	N	4	0	3a	0	0	0	TNBC	60	97	0	60	180
5*	39	9	Inv Ca NST	3	3	3	3	N	3	1a	3a	0	0	0	TNBC	280	65	85	20	0
6*	40	3.6	Inv Ca NST	3	3	3	3	N	2	0	2a	0	0	0	TNBC	75	70	0	20	210
7	56	2.2	Inv Ca NST	3	3	2	3	N	2	0	2a	0	0	0	TNBC	3	38	60	80	20
8*	39	2.5	Inv Ca NST	3	3	3	3	N	2	0	2a	0	0	0	TNBC	140	60	50	40	0
9	53	2.4	Inv Ca NST	2	3	3	3	N	2	0	2a	0	0	0	TNBC	4	72	99	10	0
10	44	1.6	Inv Ca NST	3	3	3	3	N	1c	0	1a	0	0	0	TNBC	6	62	0	10	30
11*	45	1.5	Inv Ca NST	3	3	3	3	N	1c	0	1a	0	0	0	TNBC	60	85	0	30	0
12	74	4.8	Inv Ca NST	3	3	3	3	N	2	0	2a	0	0	0	TNBC	6	40	5	270	270
13	43	2.8	Inv Ca NST	3	3	3	3	N	2	0	2a	0	0	0	TNBC	6	95	97	50	80
14*	51	2.1	Inv Ca NST	3	3	3	3	N	2	0	2a	0	0	0	TNBC	80	99	99	20	30
15	59	1.9	Inv Ca NST	3	3	3	3	N	1c	0	1a	0	0	0	TNBC	6	50	40	80	240
16*	47	2.1	Inv Ca NST	3	2	3	3	N	2	0	2a	1	0	0	Luminal A	15	75	0	250	120
17*	42	2.9	Inv Ca NST	3	3	3	3	P	2	1a	2b	0	0	0	TNBC	160	92	0	180	40
18	60	6.8	Inv Ca NST	3	3	3	3	P	3	0	2b	0	0	0	TNBC	4	82	95	300	120

*Inv Ca NST* invasive carcinoma of no special type, *TD* tubular differentiation, *NP* nuclear pleomorphism, *MC* mitotic count, *LVI* lymphovascular invasion, *P* present, *N* not identified, *T* T stage, *N* N stage, *ER* estrogen receptor, *PR* progesterone receptor, *TNBC* triple-negative breast cancer, *H-scores* histoscores.

*Indicates LGR5-positive breast cancer with histoscores 10 or higher.

**Table 2:** Association between *LGR5* positivity and the clinicopathological characteristics.

Characteristic	Total (%)	*LGR5*		*P-value*^*#*^
		Negative (%)	Positive (%)	
Patients	279 (100)	270 (97)	9 (3)	
**Age**				
≥ 55	155 (55)	147 (95 )	8 (5)	0.041
< 55	124 (45)	123 (99)	1 (1)	
**Histology**				
Invasive ca NST	250 (90)	241 (96)	9 (4)	0.898
Mucinous ca	11 (4)	11 (100)	0 (0)	
Tubular ca	4 (1)	4 (100)	0 (0)	
Invasive lobular ca	7 (3)	7 (100)	0 (0)	
Others	7 (3)	7 (100)	0 (0)	
**Tubular differentiation**				
1	24 (9)	24 (100)	0 (0)	0.105
2	66 (24)	67 (99)	0 (0)	
3	188 (67)	179 (95)	9 (5)	
**Nuclear pleomorphism**				
1	30 (11)	30 (100)	0 (0)	< 0.001
2	166 (59)	165 (99)	1(1)	
3	83 (30)	75 (90)	8 (10)	
**Mitosis**				
1	109 (39)	109 (100)	0 (0)	< 0.001
2	84 (30)	84 (98)	0 (0)	
3	86 (31)	77 (90)	9 (11)	
**Grade**				
1	44 (16)	44 (100)	0 (0)	0.001
2	131 (47)	131 (100)	0 (0)	
3	104 (37)	95 (91)	9 (9)	
**Lymphovascular invasion**				
Absent	218 (78)	210 (96)	8 (4)	0.428
Present	61 (22)	60 (98)	1 (2)	
**T stage**				
1	123 (44)	121 (98)	2 (2)	< 0.001
2	133 (48)	128 (96)	5 (4)	
3	22 (8)	21 (96)	1 (4)	
4	1 (0)	0 (0)	1 (100)	
**N stage**				
0	176 (63)	169 (96)	7 (4)	0.748
1	80 (29)	78 (98)	2 (2)	
2	15 (5)	15 (100)	0 (0)	
3	8 (3)	8 (100)	0 (0)	
Tumor stage*				
I	94 (34)	92 (98)	2 (2)	0.545
II	152 (54)	147 (97)	5 (3)	
III	33 (12)	31 (94)	2 (6)	
**ER**				
Negative	70 (25)	62 (89)	8 (11)	< 0.001
Positive	209 (75)	208 (99)	1 (1)	
**PR**				
Negative	98 (35)	89 (81)	9 (19)	< 0.001
Positive	181 (65)	181 (100)	0 (0)	
**HER2**				
Negative	224 (80)	215 (96)	9 (4)	0.131
Positive	55 (20)	55 (100)	0 (0)	
**Subtype**				
Luminal A	187 (67)	186 (99)	1 (1)	< 0.001
Luminal B	32 (12)	32 (100)	0 (0)	
HER2	22 (8)	22 (100)	0 (0)	
Triple Negative	38 (14)	30 (79)	8 (21)	

*Ca* carcinoma, *NST* no special type, *ER* estrogen receptor, *PR* progesterone receptor.

^#^Pearson chi-square test.

*AJCC 7th edition.

### Associations of *LGR5* with EGFR, CK5/6, Ki-67, and P53

**Figure 4:**
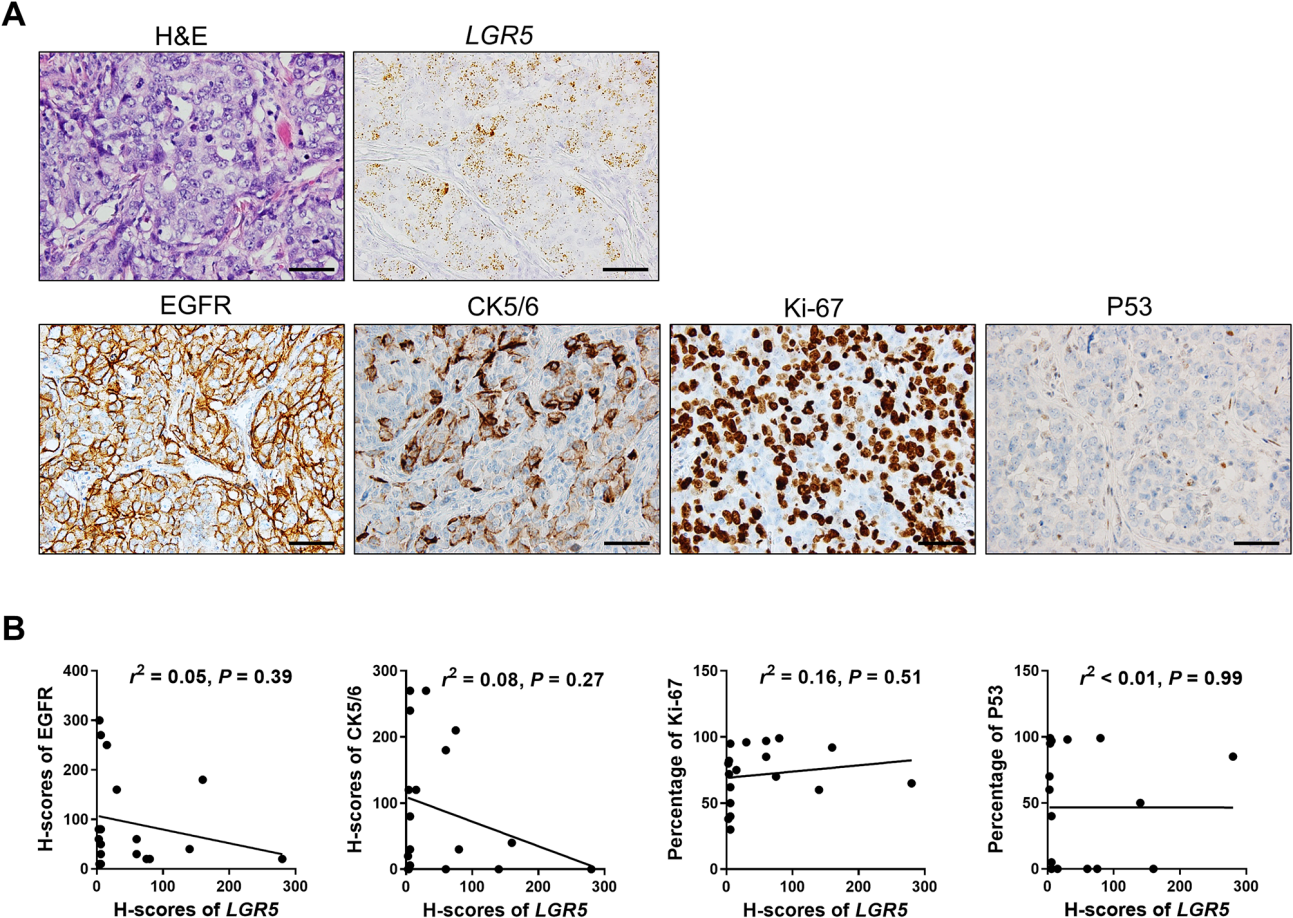
*LGR5* Expression in Triple Negative Breast Cancers. (**A**) Representative images of *LGR5* expression of a triple negative breast cancer, showing high expression for EGFR, CK5/6, and Ki-67, but negativity for P53. (**B**) Scatter plots showing the correlations of *LGR5* H-score with EGFR, CK5/6, Ki-67, and P53 expression in *LGR5*-positive BCs (*n* = 18). Scale bars: 100 μm.

The TNBC subgroup was first revealed by microarray-based expression profiling studies^25^. They are known to have particular pathological and molecular characteristics besides the lack of ER, PR, and HER2 expression: high histologic grade, high Ki-67 index, occasional presence of medullary or metaplastic elements, positivity for EGFR, CK5/6, and frequent *TP53* mutations^26,27^. As the vast majority of *LGR5*-positive BCs belong to TNBC, we investigated whether there are any correlations between *LGR5* expression levels and those distinct TNBC features. Representative images of an *LGR5*-positive BC showing high levels of EGFR, CK5/6, and Ki-67, as well as a complete loss of P53, are presented in Fig. 4A. We measured the EGFR H-scores and CK5/6 expression, as well as the percentages of Ki-67- and P53-positive cancer cells in 18 *LGR5*-expressing BCs. However, when evaluating their correlations to *LGR5* H-scores, none of them exhibited significant associations (Fig. 4B).

## Discussion

Using RNA in situ hybridization, we thoroughly investigated *LGR5* expression in normal, benign, and malignant human breast tumors. Our study demonstrated that unlike the murine model, *LGR5*-positive cells are not present in the proximal or distal ducts of adult mammary tissues. However, this can possibly be because of the limitation of our detection method. In the mouse model, lineage tracing has been used to visualize *LGR5* expression using a fluorescent reporter protein, whereas here we used ISH to determine the expression of *LGR5* mRNA. It may be that current RNA ISH techniques are not yet sensitive enough to detect cells with very low levels of *LGR5* that might exist in human mammary tissues. Additionally, as we had obtained all breast samples examined in this study from adult patients, it remains to be evaluated whether developmental stage, hormone status during menstruation, or pregnancy have any influence on *LGR5* expression.

In mice, *LGR5* is expressed in 2% to 3% of mammary epithelial cells and localized to the nipple region, and the vast majority of *LGR5*^+^ cells are myoepithelial cells^7,22^. Fu et al. have suggested distinct mammary stem cell subsets, proximally restricted *LGR5*^+^/Tspan8^hi^ cells in a deeply quiescent state can be activated by ovarian hormones and a separate pool of *LGR5*^+^/Tspan8^-^ cells in the distal portion of mammary trees^24^. In this study, we did not find any evidence of *LGR5*-positive resident stem cells in the human breast. Instead, *LGR5*-positive myoepithelial cells were observed in the scar caused by previous needle biopsies and in an inflamed tissue area formed by excision. This finding is consistent with a previous report showing that *LGR5*^+^ cells are efficient in reconstituting murine mammary glands^7^. In addition, a similar expression pattern of LGR5 has been most recently reported in the skeletal muscle regeneration. Leung et al. have shown that *LGR5* is not expressed in the satellite cells of uninjured muscle, however, it is upregulated in myogenic progenitor cells after skeletal muscle injury and *LGR5*^+^ cells contribute to muscle regeneration and satellite cell pool replenishment^28^. Therefore, it seems that in human mammary tissues, *LGR5* cells are recruited to function as facultative stem cells responsible for tissue renewal following injury. Further study is required to confirm that *LGR5* cells are a response to stem cell population to tissue damage in the human breast. An example of this is using the in vitro breast organoid system to investigate *LGR5* expression during regeneration following epithelial cell damage.

In contrast to the absence of *LGR5* expression in normal mammary tissues, it is surprising to find that *LGR5* cells are frequently detected in DCIS attenuated basal myoepithelial cells. (Fig. 1B and Supplementary Fig. S1). The mammary myoepithelial cells are involved in mammary gland development and normally facilitate milk expulsion during lactation. Studies suggest that myoepithelial cells play a tumor suppressive function by secreting various proteins such as maspin, p63, Wilms tumor 1, and laminin 1^29–31^. With DCIS progression, myoepithelial cells surrounding them become flat and are gradually lost, resulting in the transition from preinvasive to invasive cancer^32^. Therefore, it is reasonable to speculate that the appearance of *LGR5*-positive cells in the DCIS myoepithelium can be attributed to them sensing the pressure of an increasing number of cancer cells as a signal of tissue injury. Likewise, we also observed a dramatic increase in the number of *LGR5* cells in the myoepithelium of nontumorous ducts entrapped by invasive cancer cells (Supplementary Fig. S2). Alternatively, the induced *LGR5* expression in the myoepithelium might be the consequence of intricate interactions between the myoepithelium and cancer cells.

In a large cohort, we found that a total 3% of invasive BC are positive for *LGR5*. Comparing this to our previous studies using the same RNA ISH technique, the positivity is similar to that in gastric cancers (7%)^33^ and much lower than that in colorectal cancers (68%)^34^. Overall *LGR5* cancer positivity is generally associated with the basal *LGR5* expression levels in each organ, as the stomach and breast show very little or no *LGR5* expression in normal tissues, whereas the colorectum has a greater number of *LGR5* cells at the base of the crypts. Considering that *LGR5* cells are the origin of cancers in the stomach^10,12^ and colorectum^11^, its low positivity in BC is probably because of the scarcity of *LGR5* cells in the homeostatic state. More interestingly, *LGR5* positivity was exclusively observed in the TNBC subtype. Although there was one luminal A-BC that was positive for *LGR5*, its Allred score for ER was 3, and it showed high levels of CK5/6 expression, suggesting that it also harbors TNBC features. This is consistent with the previous finding from the analysis of BC samples in METABRIC database, where *LGR5* mRNA expression is significantly higher in TNBC as compared to luminal A, luminal B, and HER2 subtypes^19^. Because the expression of *LGR5* was observed exclusively in basal myoepithelial cells under regenerative conditions, it is probable that those reappearing *LGR5* cells might represent the cells of BC’s origin of the TNBC subtype.

TNBC that accounts for 10% to 20% of all BC is a highly diverse group simply defined by the absence of ER/PR/HER-2. For better molecular-based targeted therapies, there have been efforts to identify the subtypes in TNBC. For instance, Lehmann et al. suggested six different molecular subtypes of TNBC through genomic-wide gene expression profiling analyses^35^. More recently, four stable TNBC subtypes characterized by the expression of molecular profiles with distinct prognoses have been described by Burstein et al.: luminal androgen receptor, mesenchymal, basal-like immunosuppressed, and basal-like immune-activated (BLIA).^36^. Even though *LGR5* was not identified as one of the biomarkers that define subgroups in the above-mentioned studies, it would be interesting to investigate to which subtype *LGR5*-positive TNBC belongs. This would contribute to a better understanding of the molecular characteristics of *LGR5*-positive BC.

*LGR5*-positive cells have been shown to be the cancer stem cells responsible for tumor growth and metastasis in CRCs^15,16^. Yang et al. have suggested that in BC, *LGR5* + cells promote cancer cell mobility, tumor formation, epithelial-mesenchymal transition, as well as stemness by activating Wnt signaling^17^. More recently, Hagerling et al. showed a role for *LGR5* in tumor initiation in TNBC through different lineage-tracing experiments that revealed a therapeutic potential of anti-*LGR5* to target *LGR5* + cells in an aggressive ER-negative BC^19^. Although we did not continue to investigate the functional implication of *LGR5* in TNBC, we discovered an absence of *LGR5* + cells in the normal mammary tissues and specific *LGR5* expression in TNBC subtypes. These findings suggest that they would be less likely to have side effects on normal breast tissue while anti-*LGR5* therapy exerts its effects on cancer cells.

As one of the Wnt target genes, *LGR5* expression has been associated with abnormally enhanced Wnt signaling in many different types of cancers. We previously showed the positive correlations between *LGR5* and nuclear ß-catenin expression in gastric^33^ and colorectal cancers^34^. For BC, nuclear ß-catenin was reported mostly in TNBC, although *CTNNB1* mutations were not identified^37,38^, suggesting the implication of Wnt pathway activation in TNBC. However, no TNBC identified in this study displayed nuclear ß-catenin expression. Representative images are shown in Supplementary Fig. S3. This discrepancy might be because of the small number of TNBC cases in our study or in the differences in the BC patient cohort. Different criteria for nuclear ß-catenin positivity between studies might have resulted in contrary results. Further study is needed to find out whether activated Wnt signaling is involved in *LGR5* expression in TNBC or if signaling pathways other than canonical Wnt signaling are responsible for *LGR5* induction.

In summary, *LGR5* cells are not normally found in the adult human breast. However, they appear in regenerative conditions such as tissue injury, degeneration by DCIS, or entrapment by cancer cells in the mammary duct myoepithelium. This myoepithelium-restricted *LGR5* expression may be related to the specific and frequent *LGR5* expression in invasive BC of the TNBC subtype. Further studies on the functional significance of *LGR5* are required to explore *LGR5* as a potential therapeutic target for *LGR5*-positive TNBC.

## Supplementary Information

Supplementary Information.
