# Supplementary material for: Expression of LGR5 in mammary myoepithelial cells and in triple-negative breast cancers
Source: Sci Rep. 2021 Sep 7;11:17750. doi: 10.1038/s41598-021-97351-y (PMC8423726; doi:10.1038/s41598-021-97351-y)
Supplement: Supplementary file 1 — Supplementary Information. [file 41598_2021_97351_MOESM1_ESM.pdf]

Expression of *LGR5* in Mammary Myoepithelial Cells and in Triple-Negative Breast Cancers

(*Running head*: *LGR5* Expression in Breast Cancers)

Hyun Ju Lee[1], Jae Kyung Myung[2], Hye Sung Kim[3], Dong Hui Lee[7], Hyun Su Go[3], Jae Hyuck Choi[4], Hyun Min Koh[5], Su-Jae Lee[6], Bogun Jang[7]

[1]Department of Pathology, Soonchunhyang University College of Medicine and Soonchunhyang University Cheonan Hospital Cheonan, Korea
[2]Department of Pathology, Hanyang University College of Medicine, Seoul, Korea
[3]Department of Pathology, Jeju National University School of Medicine, Jeju, South Korea
[4]Department of Surgery, Jeju National University School of Medicine and Jeju National University Hospital, Jeju, South Korea
[5]Department of Pathology, Gyeongsang National Universtiy Changwon Hospital, Changwon, South Korea
[6]Department of Life Science, Research Institute for Natural Sciences, Hanyang University, Seoul, South Korea.
[7]Department of Pathology, Jeju National University School of Medicine and Jeju National University Hospital, Jeju, South Korea

Corresponding Author

Bogun Jang, MD, PhD

Department of Pathology

Jeju National University School of Medicine

Aran 13 gil 15, Jeju city, Jeju 63241, Korea

bgjang9633@gmail.com

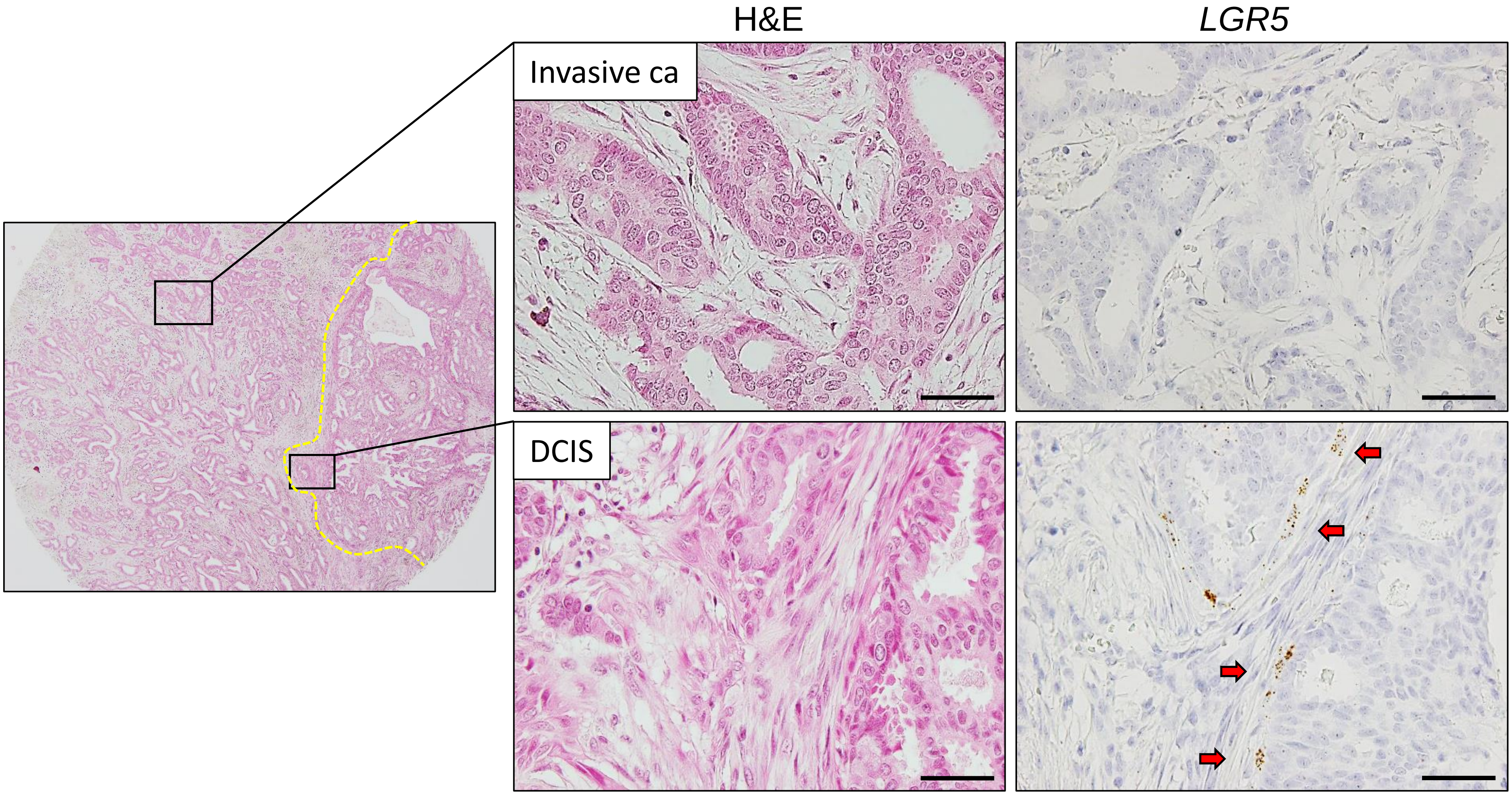

Supplementary Fig. 1 *LGR5* expression in the myoepithelial cells of ductal carcinoma in situ (DCIS). Invasive carcinoma cells express no *LGR5*, while *LGR5*-positive myoepithelial cells (indicated by red arrows) are observed in the DCIS (marked by dashed yellow line). Scale bar : 100 μm.

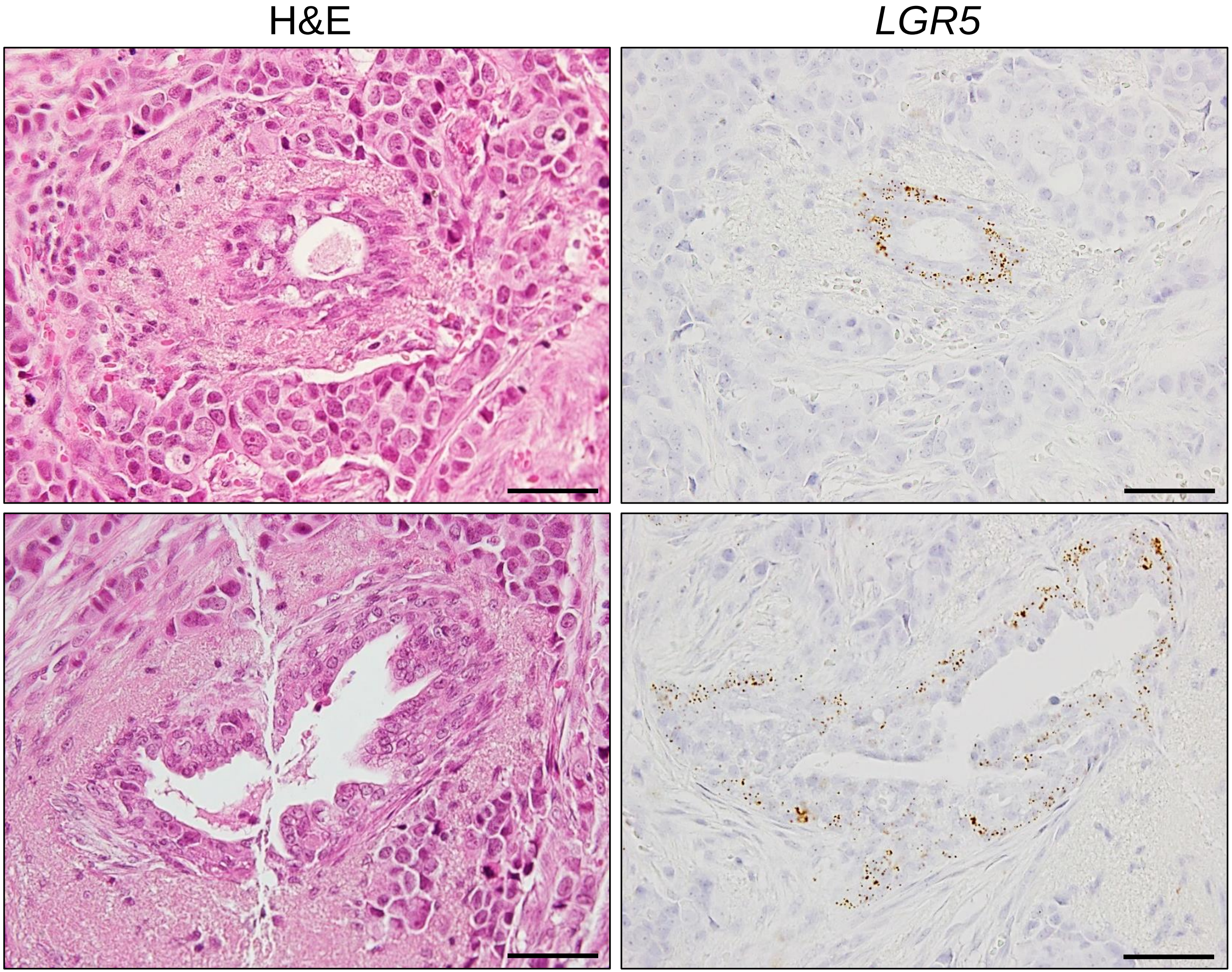

Supplementary Fig. 2 Induced *LGR5* expression in the myoepithelial cells of entrapped ducts. RNA *in situ* hybridization showed strong *LGR5* expression in the myoepithelial cells of ducts surrounded by invasive cancer cells of breast. Scale bar: 100 μm.

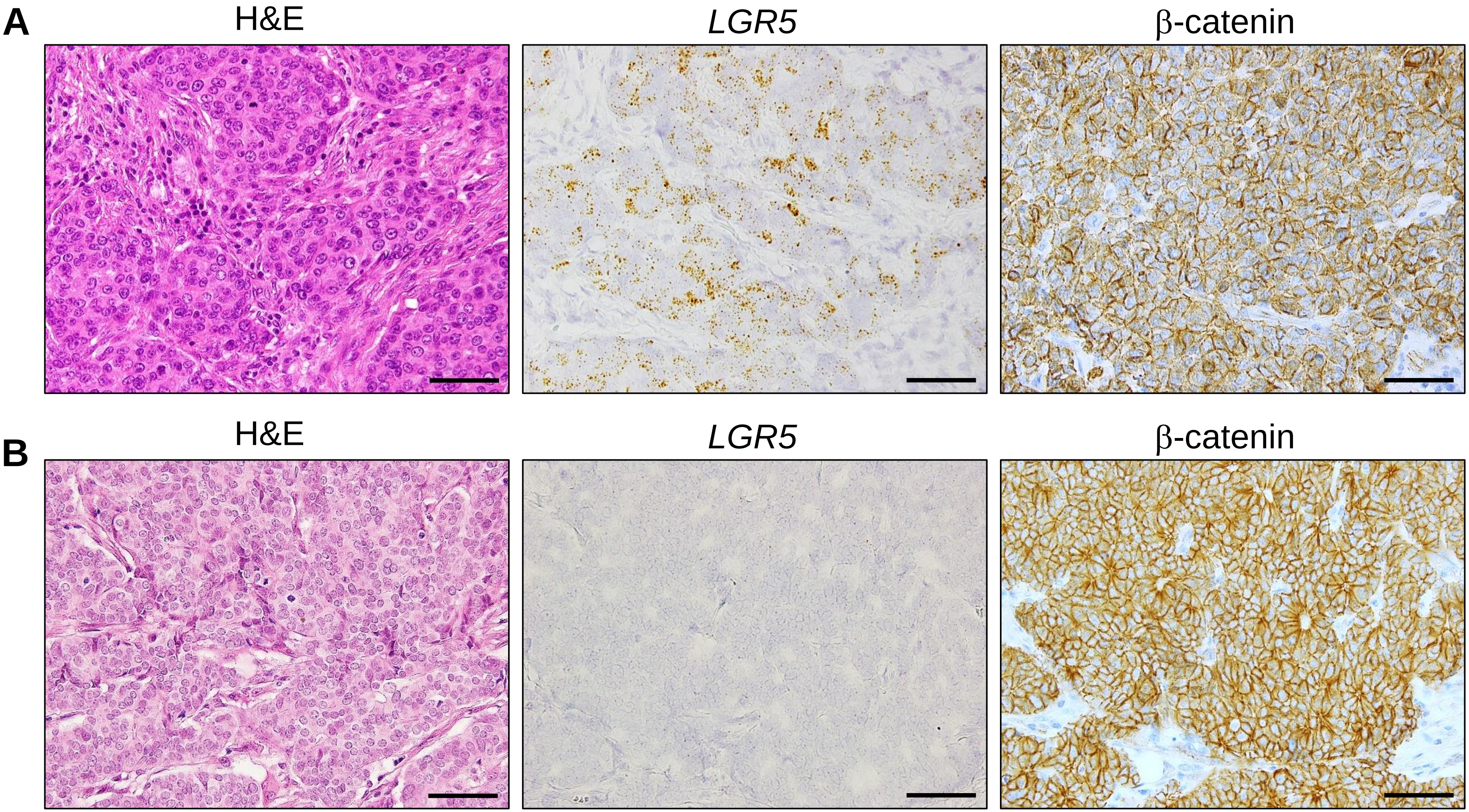

Supplementary Fig. 3 No association between *LGR5* and Wnt signaling in breast cancers. Representative images of *LGR5*-positive (A) and *LGR5*-negative (B) breast cancers and their immunohistochemical staining for β-catenin. No *LGR5*-positive breast cancers exhibited nuclear β-catenin expression. Scale bar : 100 μm.
